# Metatranscriptomic Insights Into the Response of River Biofilm Communities to Ionic and Nano-Zinc Oxide Exposures

**DOI:** 10.3389/fmicb.2020.00267

**Published:** 2020-02-26

**Authors:** Jordyn Bergsveinson, Julie Roy, Christine Maynard, Sylvie Sanschagrin, Claire N. Freeman, George D. W. Swerhone, James J. Dynes, Julien Tremblay, Charles W. Greer, Darren R. Korber, John R. Lawrence

**Affiliations:** ^1^Environment and Climate Change Canada, Saskatoon, SK, Canada; ^2^Energy, Mining and Environment Research Centre, National Research Council Canada, Montreal, QC, Canada; ^3^Department of Biology, University of Regina, Regina, SK, Canada; ^4^Canadian Light Source, Saskatoon, SK, Canada; ^5^Food and Bioproduct Sciences, University of Saskatchewan, Saskatoon, SK, Canada

**Keywords:** biofilms, microbial communities, nanomaterials, rivers, rotating annular reactors, transcriptomics

## Abstract

Manufactured Zn oxide nanoparticle (ZnO-NP) are extensively used world-wide in personal care and industrial products and are important contaminants of aquatic environments. To understand the overall impact of ZnO-NP contamination on aquatic ecosystems, investigation of their toxicity on aquatic biofilms is of particular consequence, given biofilms are known sinks for NP contaminants. In order to assess alterations in the functional activity of river microbial biofilm communities as a result of environmentally-relevant ZnO-NP exposure, biofilms were exposed to ionic zinc salt or ZnOPs that were uncoated (hydrophilic), coated with silane (hydrophobic) or stearic acid (lipophilic), at a total concentration of 188 μg l^–1^ Zn. ICP-MS analyses of biofilms indicated ZnO-NP concentrated in the biofilms, with hydrophilic, hydrophobic, and lipophilic treatments reaching 0.310, 0.250, and 0.220 μg Zn cm^–2^ of biofilm, respectively, while scanning transmission X-ray microspectroscopy (STXM) analyses of biofilms confirmed that Zn was extensively- and differentially-sorbed to biofilm material. Microbial community composition, based on taxonomic affiliation of mRNA sequences and enumeration of protozoa and micrometazoa, was not affected by these treatments, and the total transcriptional response of biofilms to all experimental exposures was not indicative of a global toxic-response, as cellular processes involved in general cell maintenance and housekeeping were abundantly transcribed. Transcripts related to major biological processes, including photosynthesis, energy metabolism, nitrogen metabolism, lipid metabolism, membrane transport, antibiotic resistance and xenobiotic degradation, were differentially expressed in Zn-exposures relative to controls. Notably, transcripts involved in nitrogen fixation and photosynthesis were decreased in abundance in response to Zn-exposure, while transcripts related to lipid degradation and motility-chemotaxis were increased, suggesting a potential role of Zn in biofilm dissolution. ZnO-NP and ionic Zn exposures elicited generally overlapping transcriptional responses, however hydrophilic and hydrophobic ZnO-NPs induced a more distinct effect than that of lipophilic ZnO-NPs, which had an effect similar to that of low ionic Zn exposure. While the physical coating of ZnO-NP may not induce specific toxicity observable at a community level, alteration of ecologically important processes of photosynthesis and nitrogen cycling are an important potential consequence of exposure to ionic Zn and Zn oxides.

## Introduction

Manufactured nanoparticle (NP) contamination of aquatic environments is of world-wide concern, as they are ubiquitously used in commonly-consumed products and present at increasingly-high concentrations in systems that include waste water treatment plant effluents, surface water, sewage sludge, and biosolids (Mueller and Nowack, 2008; Gottschalk et al., 2009; Nowack, 2009; Keller and Lazareva, 2014; Dumont et al., 2015). NPs are typically defined as materials with at least one dimension between 1 and 100 nm and exhibiting unique non-bulk properties (Handy et al., 2008a, b), with multiple factors influencing their toxicity, including total surface area, surface charge, surface reactivity, functionalization, and surface coatings (Verma and Stellacci, 2010; Zhu et al., 2010; Perreault et al., 2012). Zinc oxide nanomaterials (ZnO-NPs) are used extensively and produced at a rate of 100 to 1000 t year^–1^ (Ju-Nam and Lead, 2008; Piccinno et al., 2012). Considering that ZnO-NPs are incorporated into sunscreens and cosmetics (Schilling et al., 2010; Exbrayat et al., 2015) at content levels of up to 25%, as well as animal feeds (Sunder et al., 2007) and paints (El-Feky et al., 2014; Lu et al., 2015), along with potential for other applications (Das et al., 2011), they make a significant contribution to observed and potential environmental contamination. Given the importance of aquatic biofilms as sinks for products of nanotechnology (Battin et al., 2009), and the role of biofilm interactions with these products in terms of toxicity, fate, and effects, increased understanding of the impact of ZnO-NPs on the stability and activity of river biofilm communities is essential.

Modeling suggests that ZnO-NPs may occur in wastewater treatment plant effluents at concentrations between 0.22 and 0.74 μg l^–1^ (Gottschalk et al., 2009). Other modeling-based estimates suggest that the levels of ZnO in surface water and sewage sludge may approach 0.01 and 0.5 μg l^–1^, respectively (Mueller and Nowack, 2008; Nowack, 2009). Dumont et al. (2015) predicted that the concentration of ZnO-NPs in European surface waters was 0.1–0.5 μg l^–1^, while Keller and Lazareva (2014) predicted that biosolids could contain up to 80 mg kg^–1^ ZnO-NPs; in both studies there was a general trend to increasing concentrations.

A number of studies, including several reviews (Dizaj et al., 2014; von Moos and Slaveykova, 2014), have indicated that ZnO-NPs have toxicological activities against exposed bacteria and algae (Adams et al., 2006; Brayner et al., 2006; Franklin et al., 2007). NP toxicity is due in part to their high surface area-to-volume ratios, which may induce toxicity through interactions with a range of biological molecules, including DNA, proteins and lipids (Schrand et al., 2010). While it has been demonstrated that one important consequence of NP exposure is genotoxicity (Wang et al., 2013), toxicity may also be due to free radical production, which interferes with the integral membrane lipids and disrupts cell membranes (Reddy et al., 2007). Production of reactive oxygen species (ROS), or free metal ions, which increase in concentration with the surface area of NPs, may also generate toxicity (Kim et al., 2007; Møller et al., 2010). However, Xia et al. (2008) indicated that the toxic effects of ZnO-NPs were mainly due to particle dissolution and release of Zn^2+^, which may be taken up by the cell. This latter mechanism was also emphasized by Franklin et al. (2007) in studies comparing ionic Zn and ZnO-NPs, and concluded that the ionic forms played a significant role in metal NP toxicity on *Pseudokirchneriella subcapitata*, as did Starnes et al. (2019) following study of *Caenorhabditis elegans* response to dissolved ionic fraction. However, questions remain regarding the major sources of toxicity with regard to metal oxides with different physical properties (i.e., coatings), and ZnO-NPs in particular.

The growing potential in the application of toxicogenomic approaches for the assessment of aquatic toxicology of manufactured NPs has been noted previously (von Moos and Slaveykova, 2014). A few studies have assessed the impact of nanomaterials through the evaluation of changes in gene expression using proteomic, molecular (i.e., RT-PCR; reverse transcriptase polymerase chain reaction), fluorescent reporters, and genomic approaches. For example, Vidovic et al. (2015) examined the proteome of the bacterium *Salmonella enterica* when exposed to ZnO-NPs, which indicated a pan-metabolic effect focused on the cell membrane. While Xie et al. (2011) applied RT-PCR to assess the effects of treatment with ZnO nanoparticles on *Campylobacter jeujeni* finding up-regulation of three stress response genes. Similarly, Yu et al. (2015) applied RT-PCR to examine *amoA* gene expression levels in *Nitrosomonas europaea* grown in the presence of ZnO-NPs and observed down regulation. Su et al. (2015) used a live cell reporter assay system with a library of 1820 modified green fluorescent protein (GFP)-expressing promoter reporter vectors constructed from *Escherichia coli* K12 strains to assess gene expression during ZnO-NP exposures. These authors identified effects on specific gene with regard to expression, translation, RNA modification, and ribosome structure. Other studies on the effects of ZnO-NP and ionic-Zn on plants such as *Arabidopsis thaliana* have used transcriptomic analyses (Landa et al., 2012, 2015).

To date, transcriptomic analyses of the effects of nanomaterials have been focused on single-species exposures and evaluations, rather than metatranscriptomic analyses which may provide information of greater environmental-ecological relevance. For example, Yergeau et al. (2012) applied metatranscriptomic sequencing to river biofilm bacterial communities, demonstrating that low levels of antibiotics resulted in shifts in the relative expression of gene categories associated with ecosystem processes, including photosynthesis, carbon utilization and N- and P-metabolism, although the composition of the bacterial community was not significantly affected. Here, we examined the impact of a suite of ZnO-NPs, with and without coatings, on the metatranscriptome of river biofilm communities, without concern for specific organism-level transcriptional contributions, paralleling the approach of our previous experiments on the impacts of antibiotics on biofilm communities from the same river system (Yergeau et al., 2012). This approach was anticipated to reveal that exposure to environmentally relevant concentrations of Zn would not disturb the overall biofilm community composition, but alter the overall functional processes of biofilm communities, and induce responses directly related to toxicity, in patterns specific to the nature of the nanomaterial coating. The most profound shifts in biofilm transcriptional activity were predicted to be in response to exposure of uncoated (hydrophilic) ZnO-NP and high ionic Zn.

## Materials and Methods

### Microcosm Operation

Experimental exposures were performed in rotating annular reactors described in previous detail (Lawrence et al., 2000, 2004). Natural river water (South Saskatchewan River, Saskatoon, SK, Canada) was used as inoculum and a source of carbon and nutrients. Ionic zinc and zinc nanomaterials were added directly to individual reactors. Nutrient levels were assessed as described by Chénier et al. (2003) and the reactors were maintained at 21 + 2°C. Water was pumped through the reactors at a rate of 500 ml per day (one reactor volume) using a multichannel peristaltic pump (Watson Marlow, Wilmington, MA, United States). River biofilm communities were allowed to develop in the absence of nanomaterials for 6 weeks prior to the first exposure (94 μg l^–1^ Zn) with a second equal exposure at 7 weeks, followed by a further week of development prior to sampling. Replicated experimental treatments (*n* = 5) consisted of spiking the reactors with a total of 188 μg l^–1^ of Zn, added as hydrophobic nano-zinc (HpB), hydrophilic nano-zinc (HpL), lipophilic nano-zinc particles (LpL), and dissolved ionic-zinc (high ionic Zn; HI). In addition, one replicate group received a low concentration of ionic-zinc (LI) equal to 1.8 μg l^–1^ Zn and another replicate group received river water alone, serving as the untreated control reference condition (both groups *n* = 5). Each replicate reactor contained 12 identical biofilm coupons each 1 cm by 10 cm. After 8 weeks of biofilm development, coupons were removed from replicate reactors for immediate analysis (ICP-MS, STXM) and also flash frozen at −80°C and stored for subsequent RNA extraction and molecular analyses. All analyses (see below) were conducted on subsamples from randomly selected biofilm coupons from each replicate reactor.

### Nucleic Acids Extraction

Biofilm samples were removed from the five replicate reactor coupons for each treatment by aseptic scraping and the slurry centrifuged (5 min at ∼9000 × *g*) to separate biological material from the water phase. RNA extraction was carried out using the MoBio RNA PowerSoil kit (Hilden, Germany) following the manufacturer’s instructions. After RNA extraction, a DNAse treatment was performed using the Ambion TurboDNA free kit (Thermo Fisher, Mississauga, ON, Canada). Following the DNAse treatment, a control 16S PCR was performed to ensure that no DNA remained in the samples.

### Protozoan and Micrometazoan Enumeration

Protozoa and micrometazoa were identified based on morphology and enumerated using phase contrast microscopy. Samples were removed from the reactors on a weekly basis and the numbers of protozoa and micrometazoa manually counted on replicate 2 cm^2^ coupon subsamples. Principal component analysis (PCA) and ANOSIM testing of statistical differences (*p* < 0.05) between treatment groups (Clarke, 1993) was performed with PRIMER v6 software (PrimerE-Ltd., Lutton, United Kingdom).

### ICP-MS Analyses

Samples (one full coupon per replicate reactor) were submitted to the Saskatchewan Research Council Analytical Facility in Saskatoon, SK, Canada for extraction and determination of metal levels by ICP-MS. Analysis of differences amongst sample means for metals ICP-MS analyses carried out using the commercial package, MiniTab (State College, PA, United States), then the binary data exported and compared by PCA with PRIMER v6 software (PrimerE-Ltd., Lutton, United Kingdom).

### Scanning Transmission X-Ray Microspectroscopy and Confocal Laser Scanning Microscopy

All STXM samples were prepared by deposition of 1–5 μL of the collected biofilm material onto Si_3_N_4_ windows (1 mm × 1 mm by 100 nm thick on a 5 mm × 5 mm-sized 200 μm thick Si chip, Norcada, Inc., Edmonton, AB, Canada). Prepared STXM samples of the biofilm material on Si_3_N_4_ windows were analyzed by confocal laser scanning microscopy (CLSM) using a Nikon-C2 confocal laser microscope attached to a Nikon Eclipse 80i standard light microscope and equipped with 488/543/633 nm excitation laser sources, as well as reflection and transmission imaging (Nikon, Chiyoda, Tokyo, Japan). The fluorescent stains, Syto9 (Life Technologies, Burlington, ON, Canada) and *Triticum vulgaris* lectin-TRITC (Sigma, St. Louis, MI, United States), were used to visualize bacterial cells and exopolymer, respectively, as previously described (Neu et al., 2001). This approach also allowed the selection of representative biofilm areas that could then be systematically-analyzed using STXM.

STXM at the C 1s edge was performed on the spectromicroscopy (SM) beamline 10ID-1 at the Canadian Light Source (CLS), Saskatoon, SK, Canada (Kaznatcheev et al., 2007). The beamline was operated at an energy resolving power of E/ΔE ≥ 3000. All samples were analyzed in 1/3 atmosphere of He. The as-measured transmitted signals were converted to optical densities (OD, absorbance) using incident flux measured through regions of the window devoid of biofilm. After each analytical measurement, an image was recorded at 289 eV, an energy which readily visualizes radiation damage to polysaccharides, the most easily damaged biochemical component. The extra-cellular matrix polysaccharide signal was reduced by less than 20% as a consequence of beam damage in the worst case of the measurements reported. The microscope energy scale was calibrated to an accuracy of ± 0.05 eV using sharp gas phase signals, typically the Rydberg peaks of CO_2_. STXM was used analytically by measuring image sequences at specific energies (Jacobsen et al., 2000) or from image difference maps, which are the differences between on- and off-resonance images. Representative absorption spectra for the target species (ZnO-NP’s, protein, lipid, exopolysaccharide, CaCO_3_) were obtained by placing these species on Si_3_N_4_ windows and performing C 1s scans using STXM at the SM beamline (Lawrence et al., 2003). Component maps for the biological and nanomaterials components were obtained by fitting the measured image sequences with the reference spectra, and placed on an absolute intensity scale (OD/nm). The analysis methods have been described extensively elsewhere (Hitchcock, 2012). Data analysis was performed using aXis2000 (Hitchcock, 2014). Three or more locations (i.e., stacks) in each STXM sample were collected and quantitatively analyzed for Zn, protein, lipid, polysaccharide and CaCO_3_ content. Analyses of variance (ANOVA) were used to assess significant differences between treatments (*p* < 0.05).

### High-Throughput Sequencing

Libraries for Illumina HiSeq sequencing were prepared with the Illumina ScriptSeq Complete kit (bacteria) low input (Illumina, Inc., San Diego, CA, United States) using the protocol Lit #358-7/2013 Rev. A, which includes subtraction of bacterial ribosomal RNA. Each library was analyzed and quantified prior to sequencing with a BioAnalyzer (Bio-Rad, Hercules, CA, United States) using the High Sensitivity DNA Assay (Cat# 5067-4626, Agilent Technologies, Mississauga, ON, Canada). The pooled libraries were submitted to Genome-Quebec for paired-end sequencing (125 bp) on one lane of the HiSeq 2500 v4.

### Bioinformatics

Raw sequencing data was processed for quality, assembly, annotation, and differential expression using the SAMSA2 pipeline v2.0 (Westreich et al., 2018). Trimmomatic v0.36 was first used to process paired-end reads for quality (phred 33), using a sliding window of 4:15 and using a minimum read length of 99 bp (Bolger et al., 2014). Ribosomal reads were removed using SortMeRNA v2.1 (Kopylova et al., 2012; Supplementary Table S1) using the SILVA bacterial 16S (%ID 90) database. Processed reads (Supplementary Table S1) were used for taxonomic annotation against taxon-defining genes in MetaPhlAn2 (Truong et al., 2015), with identified reads converted to relative abundance and plotted in R v3.5.2 using ggplot2 v3.2.1 (Wickham, 2016) and viridis v0.5.1. Quality reads were subsequently functionally-annotated against NCBI’s RefSeq database (release 90; Tatusova et al., 2014) and all four hierarchical SEED subsystem databases (v2018; Overbeek et al., 2014), using DIAMOND v0.8.38 sequence aligner with flags –more-sensitive to account for variable read length, and with default e-value cutoff: 0.001, identity cut-off value of 60% and alignment length minimum of 15 (Buchfink et al., 2015). DESeq2 v3.7 was used for statistical analysis of differentially-expressed features (Love et al., 2014) between all pair-wise comparisons of experimental groups, using the geometric mean for each transcripts across all samples as means of normalization and Benjamini and Hochberg false discovery correction < 5% (*p*adj < 0.05) (Benjamini and Hochberg, 1995). ClustVis (Beta) package was used to produce PCA and heatmaps of RefSeq-annotated transcriptomes, considering the top 10 and 50% expressed transcripts, and the top 50 individual transcripts (Metsalu and Vilo, 2015). Unit variance scaling was applied and SVD with imputation used to calculate principal components (Stacklies et al., 2007). For heatmaps, rows were centered, with unit variance scaling applied, which uses the standard deviation as the scaling factor allowing for both rows and columns to be clustered using correlation distance and average linkage.

### Accession Numbers

Sequence data produced in the current study were deposited into the NCBI SRA (sequencing read archive) under Project Accession: PRJNA537135, with BioSample accession numbers SAMN11510110 through SAMN11510139.

## Results and Discussion

Bioflocs and biofilms and their associated biopolymers are the most reactive materials in aquatic systems and thus are a major potential sink for nanomaterials in the environment (Battin et al., 2009). Accordingly, it has been determined that risk assessment of metal nanomaterials based entirely on acute single-species tests may not be sufficient, and that assessments at the community level are required to understand the ecotoxicological impacts of nanomaterials (McKee and Filser, 2016). Given that the toxicity of ZnO-NPs and Zn salt has been shown to be comparable in the case of single-organism studies (Franklin et al., 2007; Aruoja et al., 2009; Peng et al., 2011) and based on the estimates of ZnO-NPs in the environment (Gottschalk et al., 2009; Keller and Lazareva, 2014) and published range of EC50 values for algae and bacteria (Bondarenko et al., 2013; Ivask et al., 2014), we selected a total sub-inhibitory exposure level of 188 μg l^–1^ (Zn) for ZnO-NPs that were uncoated (hydrophilic), coated with silane (hydrophobic) or stearic acid (lipophilic), and for ionic Zn (Zn chloride).

### Exposures

**FIGURE 1:**
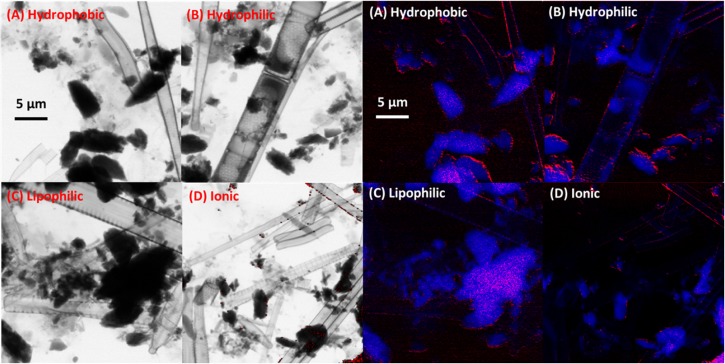
**(Left)** STXM analyses of the distribution of Zn by treatment in transmission images illustrating diatoms, bacteria, and exopolymeric substances. **(Right)** Same image series, with biological biofilm material shown in blue and Zn in red.

ICP-MS analyses of metal concentrations in the biofilm and STXM scanning transmission x-ray spectromicroscopy of the Zn-NP distribution were both used as means of assessing biofilm exposure (Lawrence et al., 2016a, b). Based on ICP-MS analyses, ZnO-NPs occurred in the biofilms with the hydrophilic, hydrophobic and lipophilic treatments, reaching 0.31, 0.25, and 0.22 μg Zn/cm^2^ of biofilm, respectively, while the high ionic exposure resulted in 0.184 μg Zn/cm^–2,^ with background levels of 0.014 μg Zn/cm^–2^ of biofilm. STXM analyses of biofilms confirmed that Zn was extensively-sorbed to biofilm materials (Figure 1). These analyses also indicated that the maximum Zn thickness (nm) for hydrophilic ZnO was 198 nm, hydrophobic ZnO was 168 nm, lipophilic ZnO was 243 nm, and high ionic was 328 nm, while average thicknesses of Zn throughout the biofilms were 9.0, 9.0, 13.0 and 14.1 nm of Zn, respectively. The visualization of Zn distribution (Figure 1) shows the extensive decoration of diatoms and apparent sorption to exopolymer within the biofilms.

**FIGURE 2:**
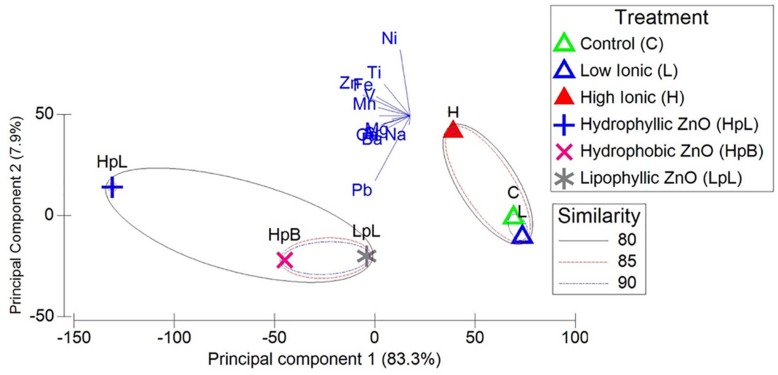
PCA of ICP-MS analysis of the concentration of metals in biofilms cultivated with South Saskatchewan River water and exposed to ionic and ZnO-NP treatments. Length of variable loadings are scaled according to R^2^ value, thus the length of each vector represents the strength of correlation or magnitude of change in the direction of each line. Prediction ellipses with probability of 0.80, 0.85, and 0.90 are presented. *N* = 30 data points.

Differences in distribution and concentration of Zn could result in microscale variation in the nature of the exposure of biofilm microorganisms, both within replicates and across experimental groups. Though this potential source of variability is in keeping with variable results observed across replicate samples in the analyses described below, the true impact of difference in metal sorption is likely negligible given that other potential contaminating metals associated with commercial nanomaterials do not effect microbial community transcriptional response (Figure 2). While it has been previously reported that adsorbed co-contaminants may cause possible so-called “Trojan horse effects,” or other indirect outcomes, for various types of nanomaterials (Jackson et al., 2013), the presence of non-Zn metals do not appear to contribute to differences in ZnO-NP treatment effect in the current study (Figure 2). The strongest association of potential contaminating metals, particularly Pb, occurs with ZnO-NP treatments. Taken together, imaging and metals analyses provide confirmation that ZnO-NPs with different physical properties and coatings are capable of interaction and concentration within river biofilms, and that under the current conditions, any observable impact on the transcriptional response from Zn-exposures are primarily due to the influence of Zn, and no other potential metal NP contaminants.

### Biofilm Community Stability

**FIGURE 3:**
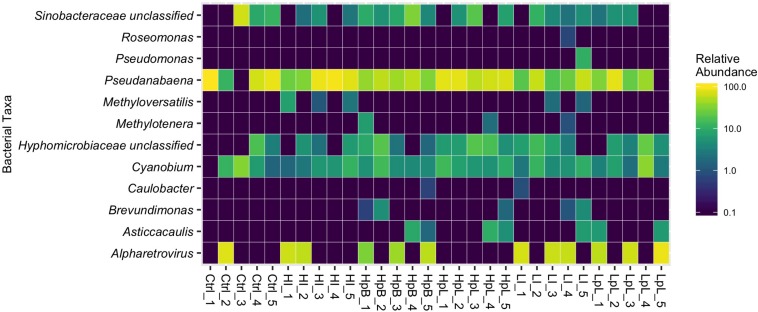
Relative abundance of biofilm microbial community organisms, with no hierarchical clustering applied. Cntrl = control; HI = high ionic Zn; LI = low ionic Zn; HpB = hydrophobic ZnO; HpL = hydrophilic ZnO; LpL = lipophilic ZnO. Numbers indicate replicate.

Analysis of community stability of the Zn-exposed and unexposed river biofilm communities from metatranscriptomic reads confirm that the biofilm community composition is stable across treatments at the sub-inhibitory concentration selected (Figure 3). The biofilms established from river water are comprised primarily by cyanobacteria including *Pseudanabaena*, followed by *Sinobacteraceae*, photosynthetic *Hyphomicrobiacea* and *Cyanobium*; with little variation in the relative abundance of these, and less-abundant taxa, between and within treatment groups. Interestingly, several samples contained relatively abundant levels of reads belonging to the *Alpharetrovirus* genus, which a recent study has found to be a common laboratory-sourced contaminant of sequencing libraries (Asplund et al., 2019). Interrogation of these viral reads did not reveal diverse viral functions, and there to be limited differential expression of viral reads across treatments, thus, reads of bacterial origin remained the primary focus of this study.

The trend of limited variation in community composition amongst experimental treatments was also observed following enumeration of protozoan and micrometazoan populations, wherein populations of these organisms were unaffected by exposure to various Zn exposures relative to the control communities (*p* < 0.05; Supplementary Figures S1, S2). This is in contrast to other studies which have reported significant effects of various stressors on protozoan population. For instance, Lawrence et al. (2012) reported that protozoa were depressed by the presence of diclofenac, whereas exposures to caffeine resulted in increased populations. Despite their documented role as ecosystem engineers (Jones et al., 1994; Mohamed et al., 1998), it appears that the interaction between Zn exposure and protozoa and micrometazoan grazing had minimal effect on overall community structure.

Despite the negligible effect of this level of Zn exposure on biofilm community composition and stability, it is an important consideration that any observed effects of ZnO-NPs on transcriptional responses at the biofilm or community level is anticipated to be far more complex, and potentially less obvious, than effects observed in the more common pure-culture study. This is for reason that relationships between organisms in polymicrobial experimentation can significantly alter the outcome of an effect, as has been found in previous studies. For example, Hendrickson et al. (2017) noted that the presence of *Streptococcus gordonii* in a consortium with *Porphyromonas gingivalis* influenced the transcription of a variety of genes by *P. gingivalis*, including increases in virulence determinants and adhesion, while stress responses were reduced. They concluded that this demonstrated an evolutionary adaptation to reduce stress when bacteria were growing in polymicrobial environments. Whether this is the case in naturally-occurring complex microbial communities would be speculative, but inter-organism interactions which maximize benefit and minimize cost (i.e., energy, toxicity) would be consistent with some models of microbial community development (Caldwell et al., 1997). As we do not use a model consortium here in favor of a naturally-established biofilm, analysis of inter-organism interactions is beyond the scope of this study, however multi-organism interaction following Zn exposure remains an important area of investigation.

### Global Effects on Gene Expression

**FIGURE 4:**
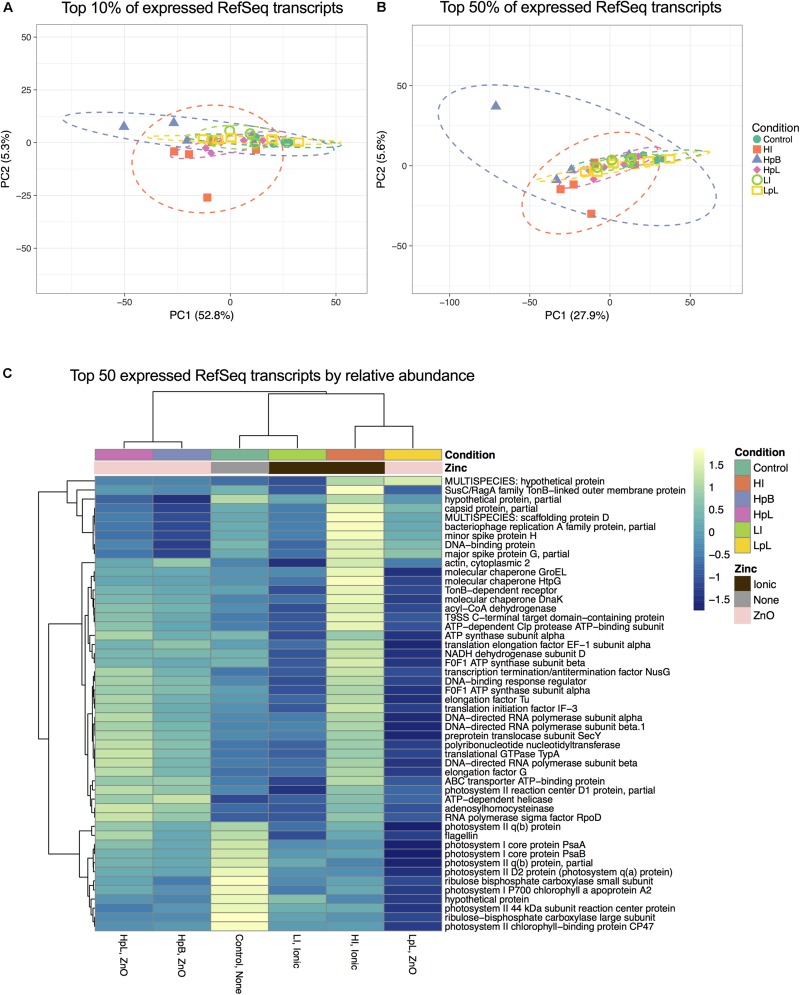
PCA analysis of RefSeq-annotated genes for (**A**) top 10% expressed genes (**B**) top 50% expressed genes across all samples. Transcripts were included for analysis if they were detected in at least three replicates; *N* = 2,548 genes for **(A)** and *N* = 8,838 genes for **(B)**. Prediction ellipses are 0.95 probability. **(C)** Heatmap of top 50 abundant individual transcripts. Read abundance is normalized for individual samples according to total sample read number, then averaged for each treatment (*n* = 5). Rows are centered with unit variance scaling applied. Both rows and columns are clustered using correlation distance and average linkage. Columns with similar annotations are collapsed by taking the mean inside each group, and the tightest cluster presented first.

Analysis of the transcriptional profile (relative abundance of RefSeq-annotated reads) of all replicates for each treatment groups by PCA revealed there to be considerable overlap in expressed transcripts (Figures 4A,B). When the top 10% abundantly expressed genes are compared to the top 50%, there is a decrease in variance explained by PC1 (52.8% variance explained amongst samples for the top 10% genes vs. 27.9% variance explained for the top 50% genes; Figures 4A,B). Thus, it is ultimately only the most highly abundant transcripts that may help explain differences between the biofilm transcriptional response to different Zn exposures. Given the dispersion or variance between replicates, it appears all treatments present some base-line influence (i.e., potential “NP-toxicity”) to the community. Thus, it may not be surprising that the majority (50%) of the transcriptional response between experimental groups is largely shared, even between control and experimental treatments (Figures 4A,B).

The widest dispersion, or greatest variability amongst replicates are found for ZnO-NP coated in silane (hydrophobic; HpB) and high ionic Zn (HI) treatments. Given the previously demonstrated toxicity of ionic Zn (Peng et al., 2011), and the presence of a hydrophobic coating which would influence the NPs possible physiological interactions with biofilm cellular components, the transcriptional response between replicates of these groups are less stable. Uncoated ZnO-NPs (hydrophilic; HpL) and stearic-acid coated ZnO-NPs (lipophilic; LpL) have tighter observation confidence intervals by comparison (Figures 4A,B), but overlap more consistently with control and very low ionic Zn treatments, indicating their physical coatings may not contribute to variation in NP interaction with, and consequent toxicity to the biofilm.

Analysis of the 50 most highly expressed transcripts suggests that there is a “core” response to Zn exposure, and that treatment-specific responses to Zn toxicity would not be observable at a global level, but rather at the level of specific transcript. Though there are small changes in the abundance of these highly expressed transcripts, it remains that their expression is shared between all experimental groups in the dataset, including controls (Figure 4C). These processes are primarily related to DNA regulation, energy and cellular maintenance (chaperones), and photosynthesis (Figure 4C), thus indicating that the biofilm community is capable of maintaining normal growth processes in face of metal toxicity and/or that the toxicity induced is not systemic. Clustering of treatment groups was done using correlation distance and average linkage, with the tightest cluster comprised of hydrophobic and hydrophilic samples (Figure 4C). Thus the means of these transcript’s expression are similar to one another between these groups, but also importantly are distinct from other clusters of control and low ionic samples, and the weak cluster of high ionic Zn treatment and lipophilic coated samples.

**FIGURE 5:**
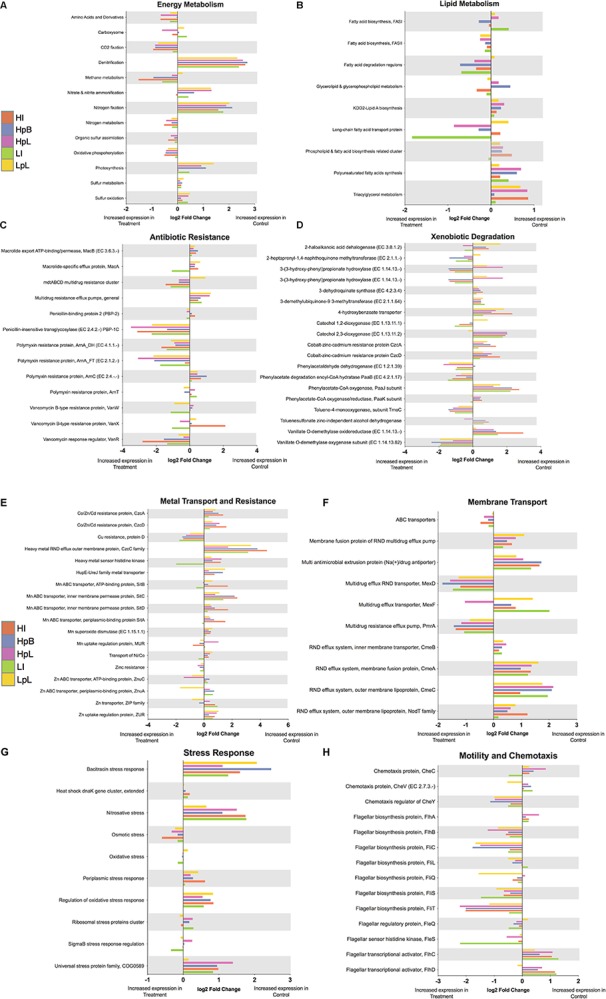
**(A–H)** Differential expression (log2 fold change) of Levels 2, 3, and 4 SEED subsystems of interest to experimental Zn treatments relative to non-Zn-treated control biofilms.

In addition to the abundant transcripts (Figure 4C), ZnO-NP and ionic treatments significantly changed the abundance of a number of transcripts relative to non-Zn-treated control biofilms (*p*adj < 0.05) related to major processes, including: photosynthesis, carbon fixation, nitrogen metabolism, amino acid metabolism, membrane transport, energy metabolism, lipid metabolism, as well as xenobiotic degradation (dioxygenases) (Supplementary Table S2). Annotation of these transcripts by the SEED subsystem classification system assisted in the parsing of cellular functions and processes that were differentially-expressed and/or abundant between treatment types (Figure 5). Differential expression (DE) analysis of transcripts grouped into SEED classifications was performed, such that pairwise comparisons between all treatment groups was calculated and the number and nature of the Level 2 SEED subsystems that experienced significant DE (sigDE; *p*adj < 0.05) across all performed comparisons, was determined. A total of 54 common cellular and/or community processes (Level 2) were subject to DE during biofilm exposure to Zn with the primary processes influenced ranging from cytochrome biogenesis, extracellular polysaccharides synthesis, iron acquisition and metabolism, membrane transport, synthesis of prosthetic groups, osmotic and periplasmic stress, and protein secretion systems (Supplementary Table S3). Processes such as general translation, ATP synthesis-coupled proton transport, and amino acid biosynthetic pathways also experienced DE during Zn exposure, as has been found for single-isolate studies involving *Salmonella enterica* serovar Enteriditis exposed to Zn-NPs (Vidovic et al., 2015). For greater understanding of what subsystem-defined transcripts were specifically influenced by different Zn exposures, DE comparison of all treatment groups to control samples at SEED subsystem Levels 2, 3, and 4 were performed.

### Energy Metabolism

Gene categories related to energy metabolism were specifically examined, including photosynthesis, carbon fixation (photosynthetic), carbon fixation (prokaryotic), sulfur metabolism, oxidative phosphorylation, methane metabolism, and nitrogen metabolism. The transcriptional profile indicates that overall photosynthetic-process (twofold), nitrogen fixation (fourfold), and denitrification (fivefold) were increased in abundance in control samples relative to all other experimental Zn treatments (Figure 5A), while cellular processes related to CO_2_-fixation, methane metabolism, nitrogen metabolism (regulation and transport), organic sulfur assimilation, and oxidative phosphorylation were all increased in abundance roughly twofold in the presence of Zn (Figure 5A). These observations fit with previous findings that concluded bacteria in the nitrogen cycle could be considered a functional group that was particularly at-risk to effects from metal-based NPs (McKee and Filser, 2016). Indeed, transcripts annotated as ammonia monooxygenase (homology to *amoA* gene), nitrite reductase (*nirK*), nitrous oxide reductase (*nosZ*), and genes related to nitrification/denitrification were present in low abundance or not detected across all Zn conditions (Supplementary Table S2), though several ammonia transporters and permeases and nitrogen regulators (subsystem: nitrogen metabolism) were, albeit at low read abundance (<0.05–0.5% total read abundance; Supplementary Table S2). This appears to parallel previous findings by Yu et al. (2015), whereby RT-PCR monitoring of *amoA* gene expression levels in *Nitrosomonas europaea*, reported that it was generally down-regulated following exposure to ZnO-NPs occurred, with He et al. (2017) further confirming ZnO-NPs inhibit nitrogen transformations through toxic effects on enzymes involved in nitrification and denitrification. While the absence of active transcription of nitrification/denitrification genes in this microbial community is interesting in the context of findings in pure culture, other factors including microbial assemblage and organism interactions may also influence the lack of nitrogen cycling and energy metabolism.

### Lipid – Fatty Acid Metabolism

Gene categories related to lipid metabolism, including biosynthesis of fatty and unsaturated fatty acids, as well as metabolism of glycerolipids, glycerophospholipids, phospholipids and triglycercols, were also assessed for effects of Zn exposure (Figure 5B). Transcripts related to fatty acid biosynthesis of (Fasl) were decreased in abundance in response to lipophilic and hydrophilic ZnO-NPs relative to controls, while variably increased in biofilms exposed to hydrophobic and high ionic Zn. In contrast, FASll-related transcripts, which would be responsible for producing a large diversity of fatty acids were consistently, but minimally (onefold) increased in abundance in response to all Zn treatments, with the greatest effect attributable to the lipophilic and hydrophilic ZnO nanomaterials (Figure 5B). Fatty acid degradation transcripts were increased in abundance in all treatments, with the exception of lipophilic ZnO-NP, while transcription of Lipid A biosynthesis-related enzymes was limited in the three different ZnO-NP treatments (Figure 4B), suggesting that bacterial cells integrity would potentially be effected by this activity in response to Zn. Indeed, there is also a decrease in transcripts related to phospholipid and fatty acid biosynthesis, polyunsaturated fatty acids synthesis, and triglycerol metabolism across ZnO-NP and high ionic Zn treatments, relative to controls (Figure 5B). Taken together, with fatty-acid degradation induced and lipid biosynthetic pathways depressed (with exception of FASII) by ZnO-NP exposure, these findings indicate potential harm to the structural integrity and lipid metabolic capacity of the biofilm community. The ability to induce controlled changes in fatty acid composition of cells as part of the general bacterial cellular envelope stress response in the presence of toxic agents in order to maintain the fluidity and symmetry of bacterial membranes is an important adaptation (Audus et al., 1992; Annous et al., 1997; Brown et al., 1997; Louesdon et al., 2015), and one that has been shown to be threatened by ZnO-NP previously in pure-culture (Vidovic et al., 2015). While different ZnO-NPs have influence on lipid metabolism to varying extents, with lipophilic and hydrophilic-coatings behaving most similarly, it is noted that all ZnO treatments induce transcriptional changes in lipid metabolism, distinct from that of experimental control samples and ionic Zn treatment.

### Antibiotic Resistance and Xenobiotic Degradation

As it is well-established that there is a close relationship between the presence of metals in the environment and the occurrence of antibiotic resistance in microbial communities (Poole, 2017), it is unsurprising that ZnO-NPs and ionic Zn both elicit transcription of antibiotic resistance pathway components, including those involved in resistance to macrolides, penicillin, polymyxin, and vancomycin (Figure 5C). Transcription of complete pathways was not uniformly observed, for example: two of the four polymyxin genes (*arnA*) were increased in Zn-exposures while *arnC* and *arnT* experienced minimal and variable transcription. In the case of penicillin resistance, a penicillin-insensitive transglycosylase gene was significantly increased (sixfold) in read abundance by all Zn treatment, whereas, a penicillin-binding protein gene was variably and minimally transcribed (Figure 5C). Similarly, the vancomycin response regulator (*vanR*) was consistently increased in read abundance (two- to fourfold) in response to Zn-exposure, while *vanX* and *vanW* exhibited no consistent pattern of transcription in response to Zn treatments (Figure 5C). Abundance of general multi-drug resistance efflux pumps was higher in control treatments; however, the *mdtABCD* multidrug resistance cluster was increased roughly twofold in all Zn treatments, with the exception of lipophilic ZnO-NP (Figure 5C).

A range of genes related to the metabolism of xenobiotic compounds were also influenced by zinc exposures (Figure 5D), though non-uniformly across treatments and within metabolic pathways. For instance, transcription of a 2-haloalkanoic acid dehalogenase was increased in response to hydrophilic and low ionic Zn exposures (roughly onefold), however were not transcribed in lipophilic, hydrophobic and high ionic Zn relative to controls biofilms (Figure 5D). Similar variability was also detected in catechol-1,2- and catechol-2,3-dioxygenase activity. Several genes exhibited a relative increase in abundance in control samples, including: 3-dehydroquinate synthase (roughly onefold), 3-demethylubiquone-9 3-methyl transferase (roughly onefold), 4-hydroxybenzoate transporter (maximum fourfold), phenylacetate-CoA oxygenase PaaJ subunit (maximum fourfold), and vanillate-*O*-demethylase oxidoreductase (roughly three- to to sixfold), suggesting a negative effect on these transcripts by Zn exposures (Figure 5C). In contrast, abundance of vanillate-*O*-demethylase oxygenase subunit (maximum fourfold), and toluene-4-monooxygenase (maximum threefold), were increased in response to all Zn exposures.

The non-uniformity of transcriptional activity for certain biosynthetic or resistance genes is due likely to the regulatory networks governing these pathways in the absence of exposure to specific antibiotics or xenobiotics, potentially constitutive expression of specific genes, and/or the effect of NPs on enzymatic activity, as opposed to noise of the experimental system. Despite the variable or incomplete transcription of certain pathways, the strong transcriptional response of specific resistance and degradative genes supports previous work in pure culture study of *Agrobacterium* sp. PH-08 exposed to Zn-NPs, which resulted in elevated levels of ROS, DNA damage, and cell death and significant reductions in biodegradation activities related to dibenzofuran and catechol (Le et al., 2014). These reductions were correlated with declines in catechol-2,3-dioxygenase activity and related gene expression, which is paralleled within the biofilm community. These observations are also in keeping with the results of Achard-Joris and Bourdineaud (2006), who found multidrug resistance proteins played a role in mercury and zinc resistance, through xenobiotic scavenging. Thus, a number of observations are consistent with both ionic- and ZnO influencing the regulation of genes/gene categories related to xenobiotic exposures.

### Metal Transport and Resistance

Among the most interesting observations is that numerous genes related to metal transport and resistance, including the cobalt-zinc-cadmium resistance protein (CzcB, CzcA, CzcD) genes and a number of Mn ABC transporter related genes, were all increased in read abundance in control biofilms relative to Zn-exposure treatments from one- to eightfold, with greatest increase in response to high ionic Zn exposure (Figure 5E). This suggest that Zn exposure at the given concentrations has a potential negative or repressive effect on some cellular resistance mechanisms. However, genes related to general Cu and Zn-resistance were transcribed to a greater extent in response all Zn-exposures, genes related to Zn transport and uptake regulation were variably-expressed and to a lesser degree in response to Zn-exposure (Figure 5E). Ultimately, while this data supports that a major source of Zn or metal NP toxicity is related to the limited transcription of metal-resistance mechanisms, this data also supports previous observations that greater toxicity results from interactions of the cell with the ionic fraction of Zn, rather than NPs (Ivask et al., 2014).

### Membrane Transport and Stress Response

Consistent with the findings of Vidovic et al. (2015), genes associated with membrane transport, and in particular, RND efflux systems, were influenced by the Zn treatments. In particular, RND systems related to general multidrug systems (*mexD* and *pmRA*) were abundance in response to Zn treatments roughly fourfold, while the multidrug resistance efflux pump (*mexF*) is only transcribed twofold in response to hydrophilic ZnO-NP exposure (Figure 5F). While these pump systems also have role in antibiotic resistance and potential xenobiotic degradation, the preference for transcription of these transport systems over the minimally transcribed ABC transporters, indicates that the hydrolysis of ATP to drive ABC transport is not an optimal energy expenditure for Zn-exposed cells, and instead cells rely on proton gradient and movement to drive transport across the membrane. The variable response of these transporters relative to other resistance transporters in Figure 5C, is exemplary of the variation observed throughout the dataset.

Variability across experimental groups is also observed when transcriptional activities related to stress response subsystems are considered (Figure 5G). Indeed, DE of general stress responses related to maintaining physiological levels of metals, cell envelop integrity, and metabolic activity surprisingly appear to be suppressed in Zn exposure (Figure 5G). For instance, the universal stress protein family COG0589 is more highly-expressed (twofold) in control biofilms, as are transcripts related to periplasmic stress response (roughly onefold), regulation of oxidative stress (roughly twofold), bacitracin stress (three- to fivefold), and nitrosative stress (two- to fourfold). While genes related to these processes are detected in the dataset (Supplementary Table S2), such as catalase and hydroperoxide reductase (i.e., dealing with oxidative stress), they are present in low overall abundance and not differentially abundant between treatment groups. It may be possible that at the Zn concentrations established here, compensatory mechanisms such as energy regulation, membrane transport, and metal resistance mechanisms are effectively used to the extent that the biofilm does not elicit a complete or targeted stress response. Indeed, the only specific stress response that has a weakly increase in related transcript abundance in response to ZnO-NP, is that of osmotic stress. Ultimately, the overall community level response observed here, in terms of maintenance of membrane stability and activation of general stress response and transporter systems, appear in keeping with those reported for pure-culture studies with bacteria (Vidovic et al., 2015).

### Motility and Chemotaxis

Interestingly, transcriptomic analyses indicated that many genes related to flagellar biosynthesis were transcribed in response to all Zn treatments one- to fourfold (Figure 5H), as well as the chemotaxis regulator *cheY* (one- to twofold). In contrast, abundance of chemotactic proteins *cheC* and *cheV* were variable, but generally increased in control biofilms. Notably, the transcriptional activators *flhC* and *flhD* were transcribed within the control samples one- to two-fold higher relative to Zn-treatments, indicating that in the absence of Zn toxicity, genes related to flagellar assembly were closely regulated, likely as a means of cellular energy-regulation (Figure 5H). In a number of cases (*cheY*, *fihB*, *fliC*, *fliT*) the greatest effects were evident in the ZnO-NP treatments (HpB, HpL, LpL), indicating that one potential outcome of the toxicity of these exposures is biofilm dissolution and/or disassociation of organisms. Further, the induction of chemotactic abilities could have significant effects on biofilm recruitment, inter-species interactions, as well as metabolic activities. Ortega-Calvo et al. (2011) reported on the effects of silver nanoparticles on the tactic response of *Pseudomonas putida* G7, and found a negative tactic response with cells becoming repelled at 100 μg l^–1^ of the NPs, but not to similar concentrations of silver nitrate. This finding would be consistent with the existence of unique toxicity mechanisms for the NP versus ionic form of the metal, and such repellent responses could certainly impact cell interactions and biofilm/community development.

## Conclusion

Sub-inhibitory and environmentally-relevant exposures of ZnO-NP and ionic Zn to photosynthetic river biofilm communities alter the abundance of genes important to cellular processes related to photosynthesis, nitrogen cycling, degradation and biosynthesis of lipids and elements of antibiotic-resistant and xenobiotic-degrading pathways. While these metabolic activities experience alterations in abundance, the biofilm community appears capable of maintaining normal cellular processes overall, and avoids eliciting specific stress responses, potentially in favor of smaller-scale compensatory activities such as metal transport, and alteration to membrane integrity and transport. Interestingly, exposure to ZnO-NP toxicity may induce a dissolution effect on biofilm, given the abundance of motility-related genes in Zn-exposures. Both ZnO-NP and ionic Zn elicit transcriptional changes in the biofilm community, with neither form causing a demonstrably more toxic effect, and often influencing cellular processes in a similar manner. Further, ZnO-NP coatings do not uniformly affect cellular processes, indicating some specific influence of surface coating on potential toxicity, with lipophilic ZnO-NPs having the weakest and most variable effect, similar to that of low ionic Zn exposure. This data supports the expectation that NP coatings and functionalization may alter the nature of these contaminants interaction with organisms in aquatic environments and indicates opportunity for assessing long-term biofilm stability in response to ZnO-NPs, along with targeted study of ZnO-NP influence on photosynthesis and nitrogen metabolism in aquatic ecosystems.

## Data Availability Statement

The datasets generated for this study can be found in NCBI SRA under Project Accession: PRJNA537135.

## Author Contributions

JL, CG, and DK contributed to the conception and design of the study. JB performed analysis and interpretation of transcriptional data and contributed to the writing and editing of the manuscript. JL and DK wrote a first draft of the manuscript. GS, JT, and JR organized the data and performed statistical analyses. CM, JR, GS, and CF carried out formal analysis. SS and JD wrote sections of the manuscript. All authors contributed to manuscript revision, read and approved the submitted version.

## Conflict of Interest

JD is employed by Canadian Light Inc. The remaining authors declare that the research was conducted in the absence of any commercial or financial relationships that could be construed as a potential conflict of interest.
