# Supplementary material for: Metatranscriptomic Insights Into the Response of River Biofilm Communities to Ionic and Nano-Zinc Oxide Exposures
Source: Front Microbiol. 2020 Feb 26;11:267. doi: 10.3389/fmicb.2020.00267 (PMC7055177; doi:10.3389/fmicb.2020.00267)
Supplement: Supplementary file 1 [file Data_Sheet_1.DOCX]

**Figure S1. PCA analysis of protozoan counts from treatment biofilms.** ANOSIM analysis reveals there to be no statistical difference (p < 0.05) in protozoan numbers between any treatment groups.

**Figure S2. PCA analysis of metazoans counts from treatment biofilms.** ANOSIM analysis reveals there to be no statistical difference (p < 0.05) in metazoans numbers between any treatment groups.
